# Cortical hemodynamic responses to acupuncture at Lianquan (CV23) in patients with post-stroke dysphagia: a functional near-infrared spectroscopy study

**DOI:** 10.3389/fneur.2026.1921352

**Published:** 2026-09-09

**Authors:** Huade Chen, Yiqun Liu, Yuying Tang, Feiyu Nong, Yalin Huang, Chongwu Xiao, Yaobin Long

**Affiliations:** 1 Department of Rehabilitation Medicine, The Second Affiliated Hospital of Guangxi Medical University, Guangxi Medical University, Nanning, China; 2 The Second Clinical Medical College of Guangxi Medical University, Guangxi Medical University, Nanning, China

**Keywords:** acupuncture, *deqi*, functional near-infrared spectroscopy, Lianquan (CV23), post-stroke dysphagia

## Abstract

**Introduction:**

Acupuncture at Lianquan (CV23) is an effective intervention for post-stroke dysphagia (PSD); however, the central neural regulatory mechanisms remain unclear. This study used functional near-infrared spectroscopy (fNIRS) with *deqi* assessment to compare cortical activation during acupuncture at CV23 between PSD patients and healthy controls, and to examine the correlation between *deqi* intensity and cortical activation.

**Methods:**

In this cross-sectional study, 21 PSD patients (PSD group) and 29 healthy controls (HC group) received acupuncture at CV23. fNIRS was used to monitor hemodynamic responses in the bilateral primary motor cortex (M1), premotor cortex/supplementary motor area (PMC/SMA), dorsolateral prefrontal cortex (DLPFC), supramarginal gyrus (SMG), primary somatosensory cortex (S1), and somatosensory association cortex (SAC) throughout a pre-stimulation baseline and three needle manipulation blocks. *Deqi* intensity was assessed with a visual analog scale (VAS). In patients with left-hemisphere lesions, fNIRS channels were mirrored to the right side, so “left” and “right” denoted the contralesional and ipsilesional sides.

**Results:**

In the PSD group, oxygenated hemoglobin (HbO) concentrations during the needle manipulation period were significantly higher than those at the pre-stimulation baseline in the contralesional M1 (*p* = 0.040), contralesional SAC (*p* = 0.040), and contralesional SMG (*p* = 0.040); meanwhile, deoxygenated hemoglobin (HbR) decreased in the contralesional S1 and SMG (*p* < 0.05), whereas no significant changes were observed in the HC group. Compared with the HC group, the PSD group exhibited significantly higher HbO concentrations in the contralesional SAC (*p* = 0.012), contralesional M1 (*p* = 0.014), contralesional S1 (*p* = 0.014), contralesional SMG (*p* = 0.014), and ipsilesional SAC (*p* = 0.014). The total *deqi* score, *suan* (aching or soreness), and *zhang* (fullness/distention or pressure) were significantly higher in the PSD group (*p* < 0.05). Significant correlations were observed between *deqi* sensations and cortical activation in the PSD group, while no significant correlations were observed in the HC group.

**Conclusion:**

Acupuncture at CV23 in PSD may be associated with altered activation in the contralesional sensorimotor network (M1, S1, SAC, and SMG), and *deqi* intensity was associated with cortical activation, providing fNIRS evidence for the association between acupuncture and the central mechanisms of PSD.

## 1 Introduction

Stroke is the second leading cause of death and the third leading cause of disability-adjusted life years worldwide, with approximately 12 million new cases occurring annually (1). Swallowing is a vital physiological function required for nutrition and airway protection and depends on the coordinated activity of cortical, subcortical, and brainstem structures (2). Neuroimaging studies have shown that swallowing is regulated by a widely distributed bilateral neural network involving the primary motor cortex (M1), primary somatosensory cortex (S1), supramarginal gyrus (SMG), insula, anterior cingulate cortex, prefrontal cortex, and cerebellum (3). Following stroke, damage to swallowing-related brain regions or their associated neural pathways can result in post-stroke dysphagia (PSD). The prevalence of PSD ranges from 37 to 78% among stroke patients (4), and it is associated with complications such as aspiration, aspiration pneumonia, and malnutrition, as well as prolonged hospitalization, increased healthcare burden, and poorer clinical outcomes (5).

Acupuncture is a key intervention in the rehabilitation of PSD. Meta-analyses have confirmed its effectiveness in improving swallowing function and reducing the risks of aspiration and subsequent aspiration pneumonia (6). Mechanistically, acupuncture is thought to act through both peripheral and central pathways: it directly stimulates local neuromuscular structures to enhance the contractile activity of swallowing muscles such as the geniohyoid, while also upregulating the excitability of swallowing-related cortical regions (7). Consistent with this, recent neuroimaging studies have demonstrated that acupuncture can specifically reshape functional connectivity within the sensorimotor network and drive compensatory reorganization of the impaired swallowing network, providing central neurobiological support for its therapeutic efficacy (8). Lianquan (CV23), located on the anterior midline of the neck above the hyoid bone, overlies swallowing-related muscles including the geniohyoid and genioglossus muscles (9) and is the most frequently used acupoint in treatment of PSD (10). The therapeutic efficacy of acupuncture at CV23 is closely linked to the induction of *deqi,* a characteristic constellation of needling sensations including soreness, numbness, distention, and heaviness (11). Systematic reviews have identified *deqi* as an important prerequisite for optimal acupuncture efficacy, with greater *deqi* intensity associated with better clinical outcomes. Accordingly, *deqi* is widely used as a key behavioral indicator for evaluating the effects of acupuncture interventions (12, 13).

Functional near-infrared spectroscopy (fNIRS) is a non-invasive neuroimaging technique that measures real-time changes in cortical hemoglobin concentrations through neurovascular coupling, thereby reflecting brain activity (14). Compared with functional magnetic resonance imaging (fMRI), fNIRS is more tolerant of head motion, safer, more convenient, and more cost-effective, making it well suited for monitoring brain activity during acupuncture interventions (15, 16). Previous fNIRS studies have shown that acupuncture can modulate hemodynamic responses within the sensorimotor network of healthy individuals (17). However, most studies have focused on a single population, and direct comparisons between PSD patients and healthy controls under a standardized acupuncture protocol remain limited. Furthermore, integrated analyses combining cortical activation patterns with multidimensional assessments of *deqi* sensation are scarce, and the neural mechanisms underlying acupuncture-induced central modulation remain incompletely understood.

Based on this background, we used fNIRS to continuously monitor cortical activation during acupuncture at CV23 in patients with PSD (PSD group) and healthy controls (HC group). Combined with multidimensional quantification of *deqi* sensation, the study aimed to compare acupuncture-induced cortical activation patterns between the two groups and examine the association between *deqi* intensity and cortical activation. We hypothesized that acupuncture at CV23 would produce distinct cortical activation patterns in PSD patients compared with healthy controls and that *deqi* intensity would be positively associated with cortical activation levels. This study may provide neuroimaging evidence for understanding the association between acupuncture and the central mechanisms of PSD, and offer a reference for exploring individualized acupuncture-based rehabilitation strategies.

## 2 Materials and methods

### 2.1 Participants

This was a cross-sectional study. The sample size was estimated using G*Power 3.1.9.7 (Kiel University, Kiel, Germany). Parameters were derived from a preliminary pilot study involving eight PSD patients and eight healthy controls in which the concentration of oxygenated hemoglobin (HbO) in the left primary motor cortex (L-M1) during needle manipulation blocks was −0.0021 ± 0.0174 mmol/L*mm for the PSD group and −0.0307 ± 0.0393 mmol/L*mm for the HC group, corresponding to an effect size of 0.939. With *α* = 0.05 (two-tailed) and power (1 − *β*) = 0.80, a minimum of 19 participants per group was required. Accordingly, 21 patients with PSD admitted to the Department of Rehabilitation Medicine at the Second Affiliated Hospital of Guangxi Medical University between June 2025 and January 2026, and 29 age- and sex-matched healthy volunteers recruited from nearby communities as the control group, were finally enrolled. The study adhered to the Declaration of Helsinki (18) and was approved by the Ethics Committee of the Second Affiliated Hospital of Guangxi Medical University (approval number: 2025-KY(0364)). All participants provided written informed consent before enrollment.

Participants were included if they fulfilled the following criteria: (1) first-ever stroke, meeting the diagnostic criteria for cerebral infarction or cerebral hemorrhage as defined in the Chinese Guidelines for Clinical Diagnosis and Treatment of Cerebrovascular Diseases (Second Edition), with confirmation of a unilateral supratentorial hemispheric lesion by cranial MRI or CT (19); (2) clinical diagnosis of PSD, with a water swallowing test (WST) Grade of 3–5 (5, 20); (3) stable vital signs, with a disease duration of 2 weeks to 12 months; (4) age 18–80 years and right-handed (8); (5) Montreal Cognitive Assessment (MoCA) score ≥26 (21); and (6) voluntarily provided written informed consent. Participants were excluded as follows: (1) non-stroke-related dysphagia; (2) severe psychiatric disorders precluding cooperation with the trial; (3) concomitant severe cardiac, pulmonary, hepatic, renal, or other organ dysfunction; (4) history of needle phobia, needle aversion, or adverse reactions to acupuncture; (5) scalp lesions, cranial defects, or other conditions preventing completion of fNIRS examination; (6) pregnant or lactating; (7) metal implants that could interfere with neuroimaging examinations; and (8) received acupuncture treatment for dysphagia or had a history of repeated acupuncture within the 3 months prior to enrollment.

### 2.2 Clinical measurements

Prior to the acupuncture intervention, the following data were collected from all participants: sex, age, years of education, body mass index (BMI), and MoCA scores, as well as lesion nature, lesion laterality, disease duration, and hemiplegia laterality for PSD patients. Swallowing function was assessed using the WST, a well-established classic screening instrument for dysphagia initially developed by Japanese scholars in 1982. Swallowing function is graded based on the time taken to consume a fixed volume of warm water and the presence of choking events. With a maximum clinical screening sensitivity of 92% and reliable diagnostic value, it has been widely implemented in clinical practice both domestically and internationally (20). Cognitive function was evaluated with the MoCA, a 30-item cognitive screening instrument with a full score of 30 that covers multiple core cognitive domains including executive function, language ability, orientation, memory recall, and visuospatial processing. Its diagnostic validity for cognitive impairment screening has been sufficiently validated (22).

### 2.3 Acupuncture manipulation and *deqi* sensation

**Figure 1:**
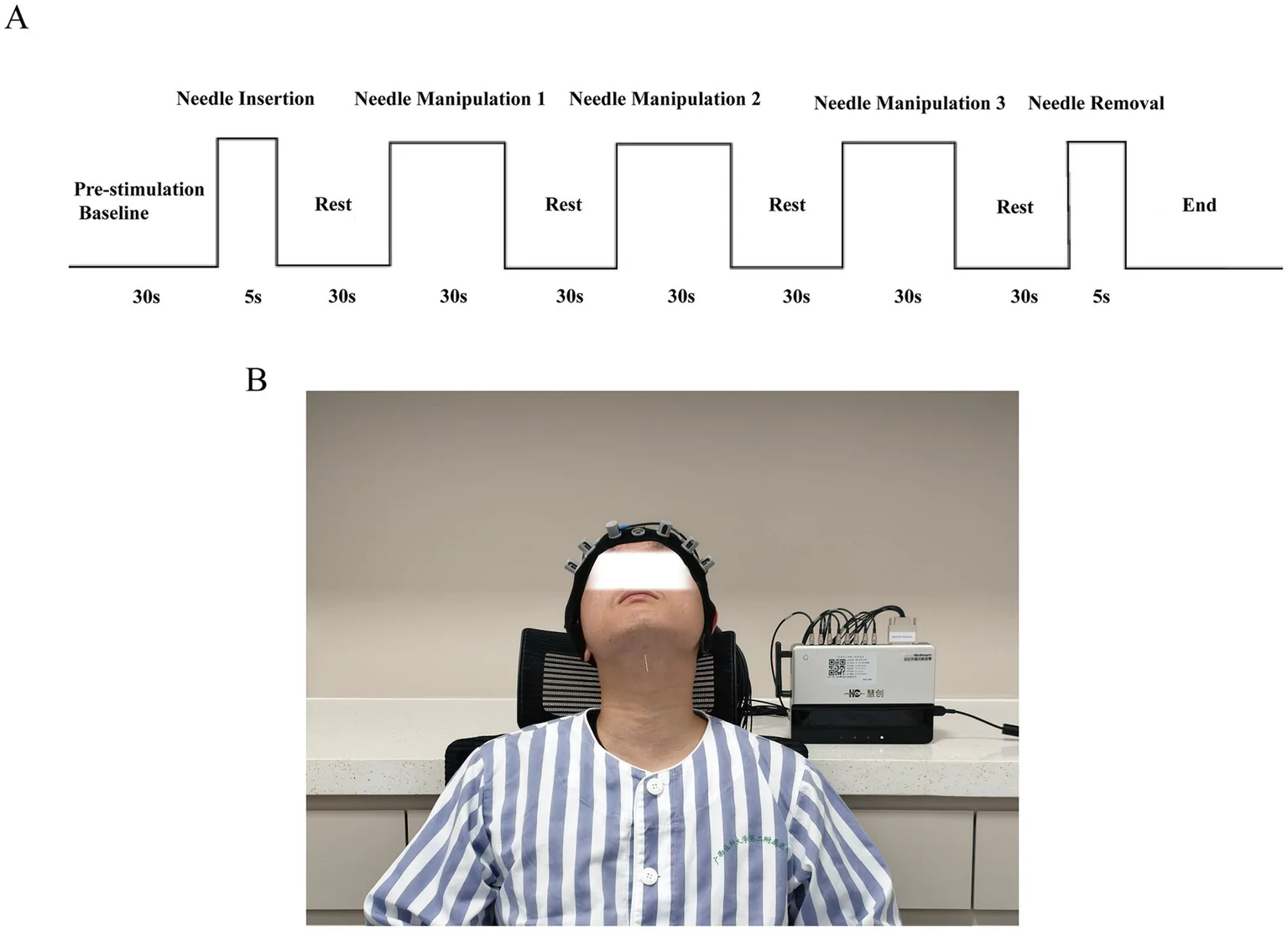
Experimental paradigm and setup. **(A)** Schematic diagram of the experimental paradigm. The paradigm consisted of a 30-s pre-stimulation baseline, needle insertion (completed within 5 s), an initial 30-s rest period, three needle manipulation blocks (30 s of bidirectional twirling stimulation followed by 30 s of rest each), and needle removal (5 s). **(B)** Illustration of a participant receiving acupuncture at CV23 while wearing the fNIRS headcap.

All acupuncture procedures at CV23 were performed by the same licensed acupuncturist with >5 years of clinical experience, using disposable sterile needles (φ 0.25 × 40 mm, Suzhou Huatuo Medical Instrument Co., Ltd., Suzhou, China). All operations strictly followed standardized protocols. The experimental paradigm consisted of a 30-s pre-stimulation baseline, followed by needle insertion (completed within 5 s) and an initial 30-s rest period. Subsequently, three needle manipulation blocks were performed, each consisting of 30 s of bidirectional twirling stimulation (depth ~15 mm, amplitude ±60°, frequency 120 times/min (23)) followed by 30 s of rest. Finally, the needle was withdrawn within 5 s (Figure 1A). Following acupuncture, the intensity of *deqi* sensation was evaluated in all participants using the visual analog scale (VAS) (24). The scale covered six types of acupuncture-related sensations, which included *suan* (aching or soreness), *ma* (numbness or tingling), *zhang* (fullness/distention or pressure), *tong* (dull pain), *zouchuan* (spreading, a feeling of extension from the acupuncture position to the surrounding area), and *zhong* (heaviness) (Supplementary Table 1) (17). Each VAS was a continuous scale ranging from 0 to 10 (0 = no sensation, 10 = unbearable intense sensation). The total *deqi* score was calculated as the sum of the scores for the six items. The VAS was completed once by each participant immediately after needle withdrawal to reflect the overall *deqi* sensation across the three needle manipulation blocks. Participants were not informed of their fNIRS results.

### 2.4 fNIRS measurement and data processing

Data were acquired using a fNIRS system (NirSmart-6000A, Danyang Huichuang Medical Equipment Co., Ltd., Danyang, China). The system employed dual-wavelength near-infrared light at 730 and 850 nm, with a sampling rate of 11 Hz. The optode array consisted of 23 emitters and 15 detectors, yielding 49 effective measurement channels. The source-detector separation was uniformly set at 3.0 cm, in accordance with international standards for adult fNIRS testing (25). The experiment was conducted in a dedicated, sound-attenuated, temperature-controlled (26 °C), softly lit examination room. Participants wore the fNIRS probe cap, sat with their head position fixed, and were instructed to keep their eyes closed, remain quiet, and strictly avoid extraneous movements such as head motion and swallowing (Figure 1B).

**Figure 2:**
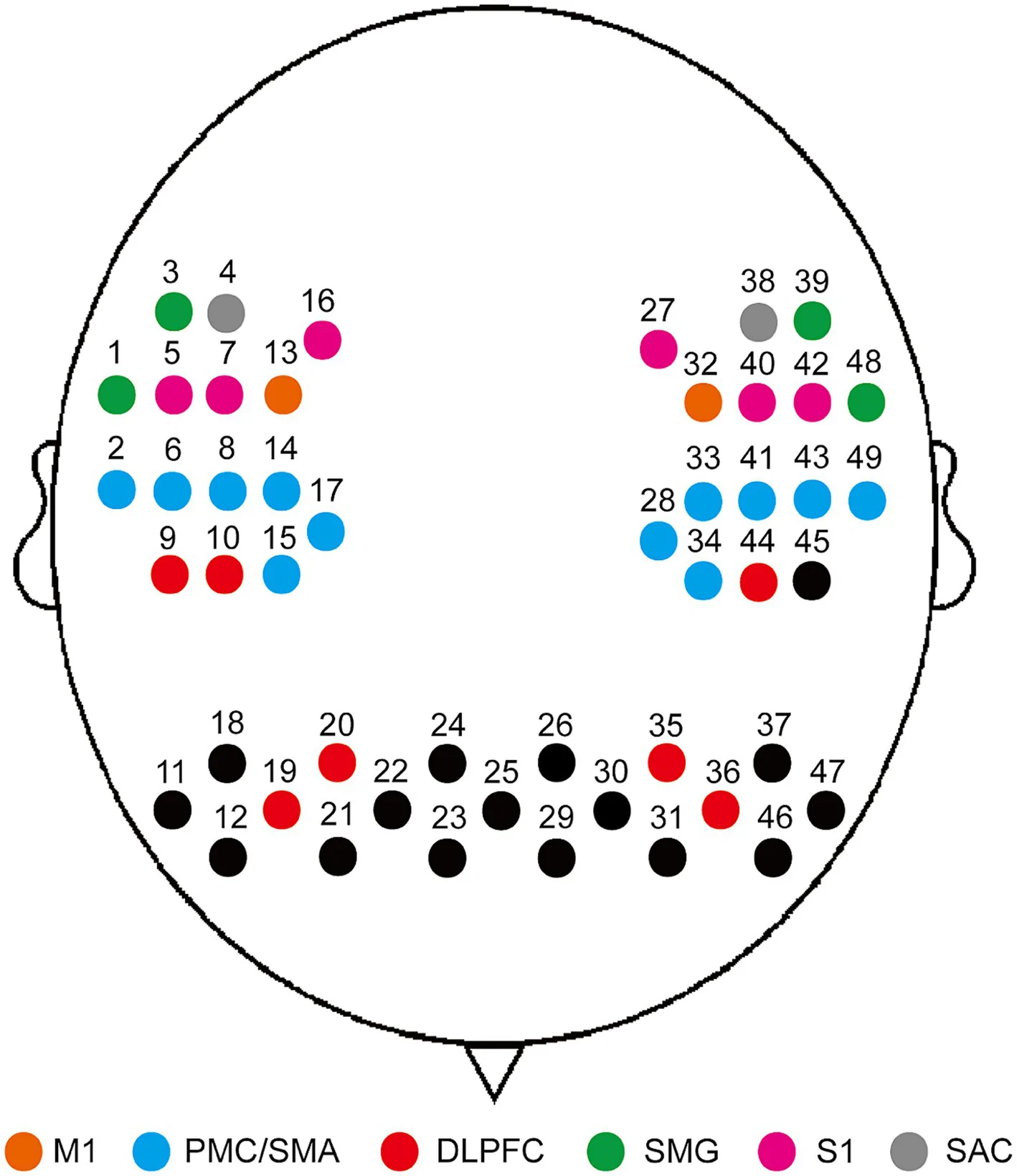
Channel layout and regions of interest (ROIs). Each solid circle denotes a measurement channel, with the channel number indicated above it. Channels are color-coded according to ROI assignment: Orange, Primary motor cortex (M1); Blue, Premotor cortex/supplementary motor area (PMC/SMA); Red, Dorsolateral prefrontal cortex (DLPFC); Green, Supramarginal gyrus (SMG); Pink, Primary somatosensory cortex (S1); Gray, Somatosensory association cortex (SAC). Black circles indicate channels not assigned to any ROI.

Spatial coordinates of all optodes relative to the International 10–20 System reference points were obtained using a three-dimensional digitizer (26). These coordinates were registered and mapped onto the Montreal Neurological Institute standard brain space, corresponding to the relevant Brodmann area (27). Based on the anatomical localization of swallowing-related brain regions and previous studies (28, 29), the following regions of interest (ROIs) were defined: L-M1, right primary motor cortex (R-M1), left premotor cortex/supplementary motor area (L-PMC/SMA), right premotor cortex/supplementary motor area (R-PMC/SMA), left dorsolateral prefrontal cortex (L-DLPFC), right dorsolateral prefrontal cortex (R-DLPFC), left supramarginal gyrus (L-SMG), right supramarginal gyrus (R-SMG), left primary somatosensory cortex (L-S1), right primary somatosensory cortex (R-S1), left somatosensory association cortex (L-SAC), and right somatosensory association cortex (R-SAC). The channel arrangement and ROI delineation are illustrated in Figure 2 and Table 1.

**Table 1:** Regions of interest and corresponding channel configuration.

Region of interest (ROI)	Abbreviation	Number of channels	Correspondence channels	Brodmann areas (BA)
Left primary motor cortex	L-M1	1	32	4
Right primary motor cortex	R-M1	1	13	4
Left premotor cortex/supplementary motor area	L-PMC/SMA	6	28, 33, 34, 41, 43, 49	6
Right premotor cortex/supplementary motor area	R-PMC/SMA	6	2, 6, 8, 14, 15, 17	6
Left dorsolateral prefrontal cortex	L-DLPFC	3	35, 36, 44	9, 46
Right dorsolateral prefrontal cortex	R-DLPFC	4	9, 10, 19, 20	9, 46
Left supramarginal gyrus	L-SMG	2	39, 48	40
Right supramarginal gyrus	R-SMG	2	1, 3	40
Left primary somatosensory cortex	L-S1	3	27, 40, 42	1, 2, 3
Right primary somatosensory cortex	R-S1	3	5, 7, 16	1, 2, 3
Left somatosensory association cortex	L-SAC	1	38	5, 7
Right somatosensory association cortex	R-SAC	1	4	5, 7

Probe placement follows the international 10–20 system.

fNIRS data preprocessing was performed using NirSpark (Danyang Huichuang Medical Equipment Co., Ltd., Danyang, China). To eliminate the confounding effect of lesion laterality on group-level analyses, the fNIRS channel layout for all patients with left-hemisphere lesions was horizontally mirrored along the midsagittal plane (30). This ensured that the lesion was uniformly mapped to the right hemisphere across all patients. Accordingly, the left and right hemispheres are consistently referred to as the contralesional and ipsilesional sides, respectively, denoted by L- and R- prefixes. HC data were not mirrored and were analyzed in their native anatomical orientation. Signal quality was assessed using the coefficient of variation (CV) of the raw optical intensity at the two wavelengths, calculated as (standard deviation/mean) × 100. Channels with a CV ≥ 15% at either wavelength in any participant were excluded from further analysis. Motion artifacts in the raw optical intensity signals were corrected using spline interpolation. A 0.01–0.2-Hz Butterworth bandpass filter was then applied to remove physiological noise, including high-frequency cardiorespiratory oscillations and low-frequency signal drift. The denoised optical intensity signals were converted to optical density, and concentration changes of HbO and deoxygenated hemoglobin (HbR) were calculated using the modified Beer–Lambert law, with the differential pathlength factor set to 6 for each wavelength (31). Given its higher signal-to-noise ratio and greater sensitivity to neural activation (25), HbO concentration was selected as the primary outcome measure. Data from the three needle manipulation blocks were averaged to represent activation during the needle manipulation period, and data from the 30-s pre-stimulation baseline were extracted to serve as the baseline activation level.

### 2.5 Statistical analysis

Data analysis was performed using SPSS 27.0 (IBM Corp., Armonk, NY) by independent researchers blinded to group allocation. Categorical variables are presented as frequencies and percentages. Continuous variables were assessed for normality by the Shapiro–Wilk test and for homogeneity of variance by Levene’s test, and are reported as mean ± standard deviation when normally distributed, or as median with interquartile range otherwise. For demographic characteristics, sex was compared using the Chi-squared test; age, years of education, BMI, and MoCA scores were compared by independent samples *t*-test if normally distributed, or by Mann–Whitney U test if not. Clinical features of the PSD group are summarized descriptively. For fNIRS data, group comparisons of HbO and HbR concentrations were conducted separately using data from the pre-stimulation baseline period and the needle manipulation period: normally distributed data with equal variances were compared using independent samples *t*-test, normally distributed data with unequal variances using Welch’s *t*-test, and non-normally distributed data using the Mann–Whitney U test, with the baseline comparison used to assess the comparability of the two groups at baseline. Within-group comparisons between the needle manipulation period and pre-stimulation baseline were performed using paired-sample *t*-test for normally distributed data and Wilcoxon signed-rank test for non-normally distributed data. *Deqi* sensation scores, assessed by VAS, were compared between groups using the same approach. Correlation analyses between *deqi* sensation scores and cortical HbO concentration were conducted separately within each group: Pearson correlation was used for normally distributed data, and Spearman rank correlation was used for non-normally distributed data. All multiple comparisons involving multiple ROIs or multiple dimensions were corrected using the false discovery rate (FDR) method, with statistical significance set at *p* < 0.05. Effect sizes for normally distributed data are reported as Cohen’s d with its 95% confidence interval (CI) calculated based on the noncentral t-distribution; for non-normally distributed data, effect sizes are reported as the rank-biserial correlation coefficient (r = |Z|/√N), with its 95% CI approximated using Fisher’s z transformation. All hypothesis tests were two-tailed.

## 3 Results

### 3.1 Baseline characteristics and comparability analysis

As shown in Table 2, no statistically significant differences were observed between the two groups in sex, age, years of education, BMI, or MoCA scores (*p* > 0.05). In the PSD group, the median disease duration was 7 months (interquartile range: 5.3–8.0). Twelve patients (57.1%) had ischemic stroke, and nine (42.9%) had hemorrhagic stroke. The WST grades were as follows: eight patients in Grade 3, eight in Grade 4, and five in Grade 5. Eleven patients had left-sided hemiplegia and 10 had right-sided hemiplegia. Signal quality assessment showed that the mean CV across all channels ranged from 1.54 to 3.92% in the PSD group and from 2.68 to 5.17% in the HC group. A total of 5 subject–channel combinations (0.72% of 693) were excluded in the PSD group, and 13 (1.36% of 957) in the HC group. No channels were entirely excluded in either group. The mean CV, range, and exclusion details for each channel at 730 nm and 850 nm are provided in Supplementary Table 2.

**Table 2:** Baseline characteristics of the participants.

Variable	HC group (*n* = 29)	PSD group (*n* = 21)	*p* value
Age (years)	54.17 ± 8.43	56.43 ± 8.78	0.363
Sex (male/female)	19/10	13/8	0.793
Education (years)	12.62 ± 1.74	12.52 ± 2.06	0.858
BMI (kg/m^2^)	23.88 ± 2.13	24.94 ± 2.25	0.096
MoCA (scores)	28.10 ± 1.29	27.71 ± 1.31	0.289
Disease duration (months)	–	7.00 (5.25–8.00)	–
Stroke type (ischemic/hemorrhagic)	–	12/9	–
WST (Grade 3/4/5)	–	8/8/5	–
Hemiplegia side (left/right)	–	11/10	–

Data are presented as mean ± SD, median (IQR), or frequency. –, not applicable for the HC group.

### 3.2 Cortical activation analysis

#### 3.2.1 Within-group cortical activation results

In the HC group, no statistically significant differences were observed in HbO or HbR concentrations in any ROI (*p* > 0.05). In the PSD group, HbO concentrations during the needle manipulation period were significantly higher than those at the pre-stimulation baseline in L-M1 (*p* = 0.040, *d* = 0.646, 95% CI [0.168, 1.112]), L-SAC (*p* = 0.040, *d* = 0.617, 95% CI [0.143, 1.079]), and L-SMG (*p* = 0.040, *d* = 0.651, 95% CI [0.172, 1.117]); no statistically significant differences were observed in the remaining ROIs (*p* > 0.05). HbR concentrations during the needle manipulation period were significantly lower than those at the pre-stimulation baseline in L-S1 (*p* = 0.042, *r* = 0.588, 95% CI [0.209, 0.813]) and L-SMG (*p* = 0.024, *r* = 0.671, 95% CI [0.337, 0.855]); no statistically significant differences were observed in the remaining ROIs (*p* > 0.05). The full within-group comparison results are presented in Supplementary Tables 3, 4.

#### 3.2.2 Between-group cortical activation results

**Figure 3:**
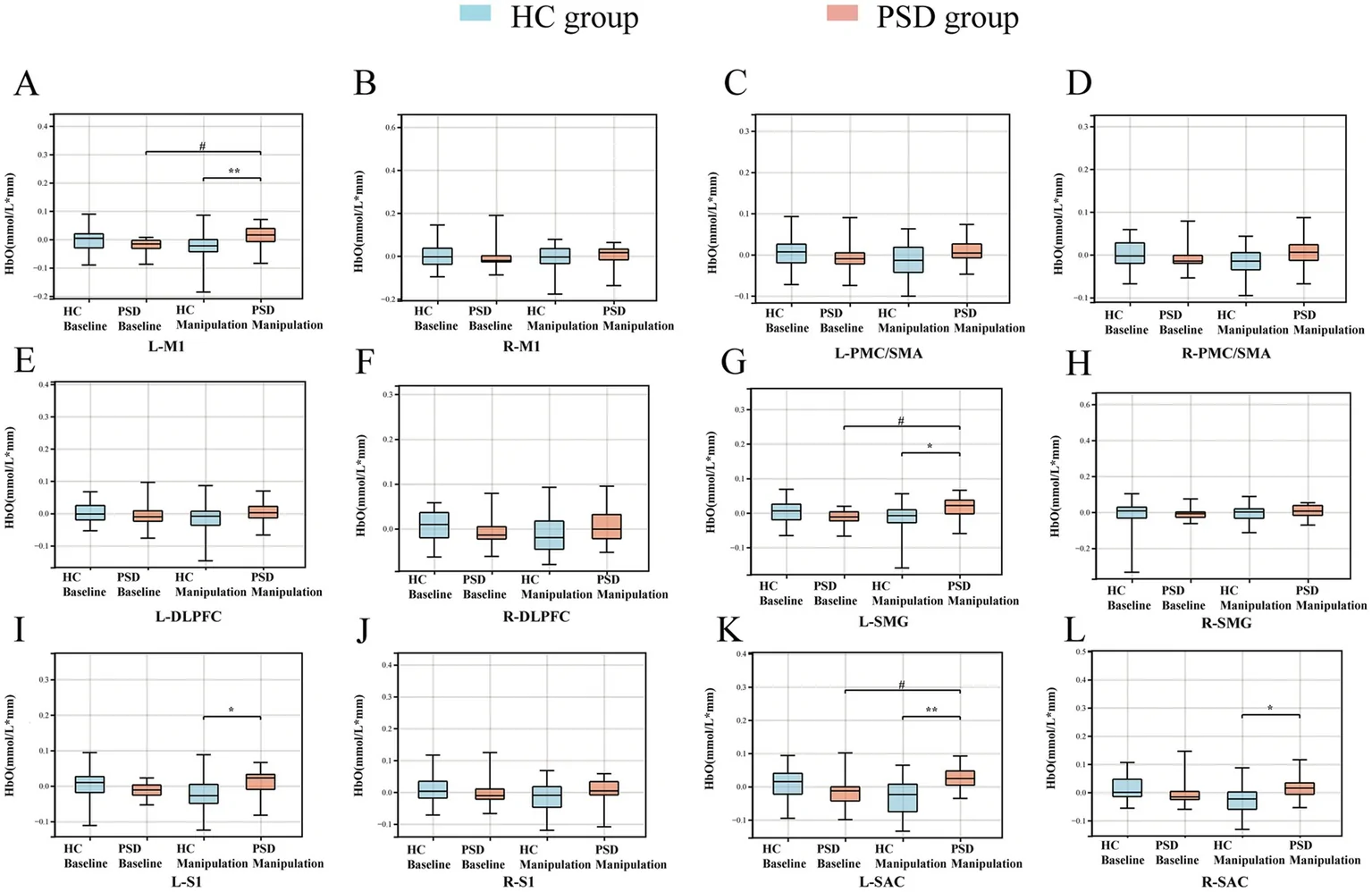
Cortical activation across all ROIs in the HC and PSD groups during pre-stimulation baseline and needle manipulation. **(A)** L-M1, **(B)** R-M1, **(C)** L-PMC/SMA, **(D)** R-PMC/SMA, **(E)** L-DLPFC, **(F)** R-DLPFC, **(G)** L-SMG, **(H)** R-SMG, **(I)** L-S1, **(J)** R-S1, **(K)** L-SAC, **(L)** R-SAC. Each ROI displays four boxes from left to right: HC during pre-stimulation baseline, PSD during pre-stimulation baseline, HC during needle manipulation, and PSD during needle manipulation. All *p*-values were corrected using the FDR method. # *p* < 0.05 for within-group comparisons; * *p* < 0.05, ** *p* < 0.01 for between-group comparisons.

**Figure 4:**
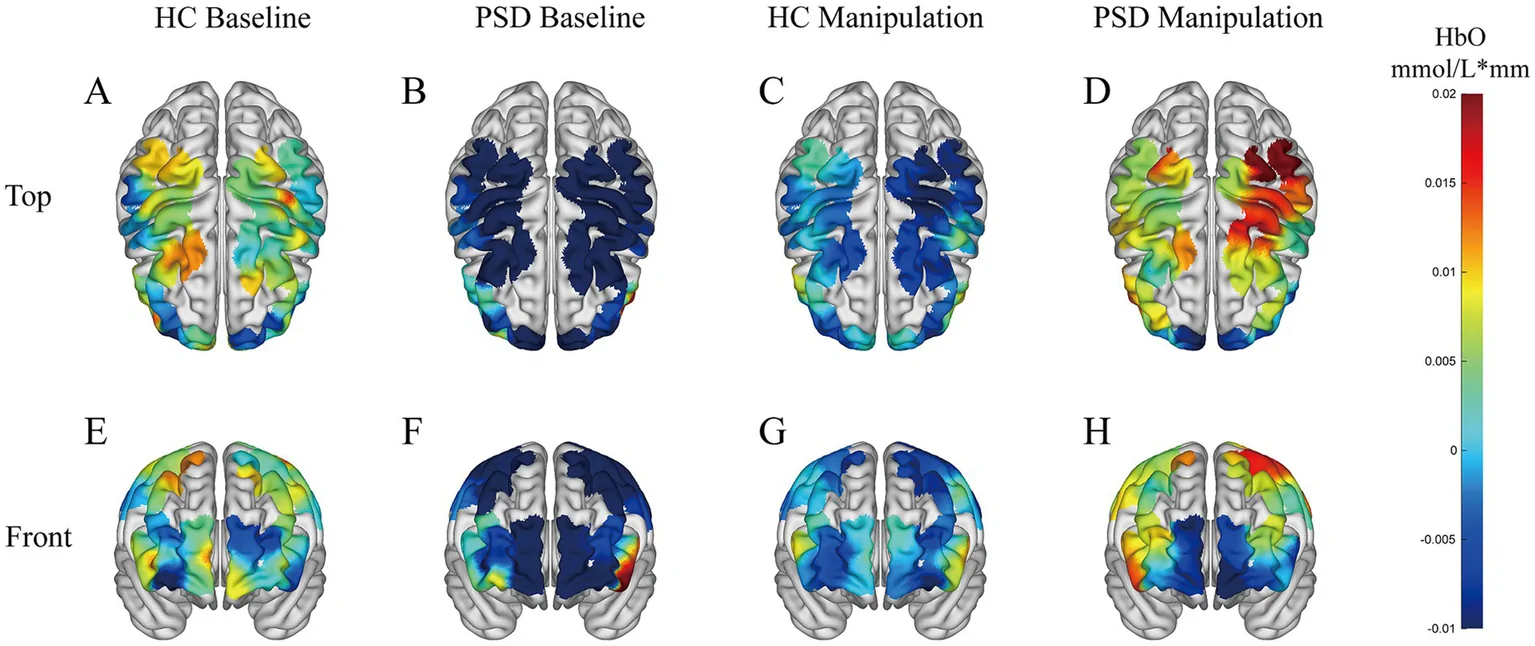
Three-dimensional cortical activation maps of the HC and PSD groups during pre-stimulation baseline and needle manipulation. Top row: top view; bottom row: front view. **(A,E)** HC group at pre-stimulation baseline; **(B,F)** PSD group at pre-stimulation baseline; **(C,G)** HC group during needle manipulation; **(D,H)** PSD group during needle manipulation. The color bar represents HbO concentration (mmol/L*mm), with red indicating higher activation and blue indicating lower activation.

No significant between-group differences in HbO concentrations were observed in any of the 12 ROIs during the pre-stimulation baseline period (*p* > 0.05), as shown in Supplementary Table 5. During the needle manipulation period, the PSD group exhibited significantly higher HbO concentrations than the HC group in L-SAC (*p* = 0.012, *d* = 1.097, 95% CI [0.489, 1.694]), L-M1 (*p* = 0.014, *d* = 0.834, 95% CI [0.244, 1.415]), L-S1 (*p* = 0.014, *d* = 0.847, 95% CI [0.256, 1.429]), L-SMG (*p* = 0.014, *r* = 0.388, 95% CI [0.122, 0.602]), and R-SAC (*p* = 0.014, *d* = 0.854, 95% CI [0.263, 1.437]). No significant between-group differences in HbR concentrations were observed in any ROI during either the pre-stimulation baseline period or the needle manipulation period (*p* > 0.05), as shown in Supplementary Table 6. Figure 3 presents the HbO concentrations and statistical comparisons for all ROIs at the pre-stimulation baseline and during the needle manipulation period in both groups. Figure 4 displays the three-dimensional cortical activation maps of the two groups. The full between-group comparison results for HbO are presented in Supplementary Table 7.

### 3.3 *Deqi* sensation analysis

#### 3.3.1 Between-group comparison of *deqi* scores

The total *deqi* score was significantly higher in the PSD group than in the HC group (*p* = 0.004, *r* = 0.532, 95% CI [0.298, 0.706]). The score for *suan* (aching) was significantly higher in the PSD group than in the HC group (*p* = 0.004, *r* = 0.572, 95% CI [0.349, 0.733]). The score for *zhang* (fullness) was significantly higher in the PSD group than in the HC group (*p* = 0.019, *r* = 0.373, 95% CI [0.105, 0.590]). No statistically significant between-group differences were found for the remaining *deqi* items (*p* > 0.05) (Table 3).

**Table 3:** Between-group comparisons of *deqi* sensation scores.

*Deqi* item	HC group (*n* = 29)	PSD group (*n* = 21)	*p* value	Effect size [95% CI]
*suan* (aching or soreness)	0.0 (0.0, 1.0)	2.0 (1.0, 3.0)	0.004**	0.572 [0.349, 0.733]
*ma* (numbness or tingling)	0.0 (0.0, 2.5)	0.0 (0.0, 0.0)	0.366	0.178 [−0.106, 0.435]
*zhang* (fullness/distention or pressure)	0.0 (0.0, 2.0)	2.0 (0.0, 3.5)	0.019*	0.373 [0.105, 0.590]
*tong* (dull pain)	0.0 (0.0, 1.5)	0.0 (0.0, 2.5)	0.367	0.159 [−0.125, 0.419]
*zouchuan* (spreading, a feeling of extension from the acupuncture position to the surrounding area)	0.0 (0.0, 2.0)	0.0 (0.0, 1.5)	0.440	0.109 [−0.174, 0.376]
*zhong* (heaviness)	0.0 (0.0, 1.0)	0.0 (0.0, 2.0)	0.412	0.131 [−0.153, 0.395]
Total *deqi* score	5.0 (3.0, 6.0)	8.0 (5.5, 9.5)	0.004**	0.532 [0.298, 0.706]

Data are presented as median (P₂₅, P₇₅). The Mann–Whitney U test was used for between-group comparisons. Effect size is reported as the rank-biserial correlation coefficient r with its 95% CI estimated using Fisher’s z transformation. All *p*-values were corrected using the FDR method. * *p* < 0.05, ** *p* < 0.01.

#### 3.3.2 *Deqi* sensation and cortical activation

**Figure 5:**
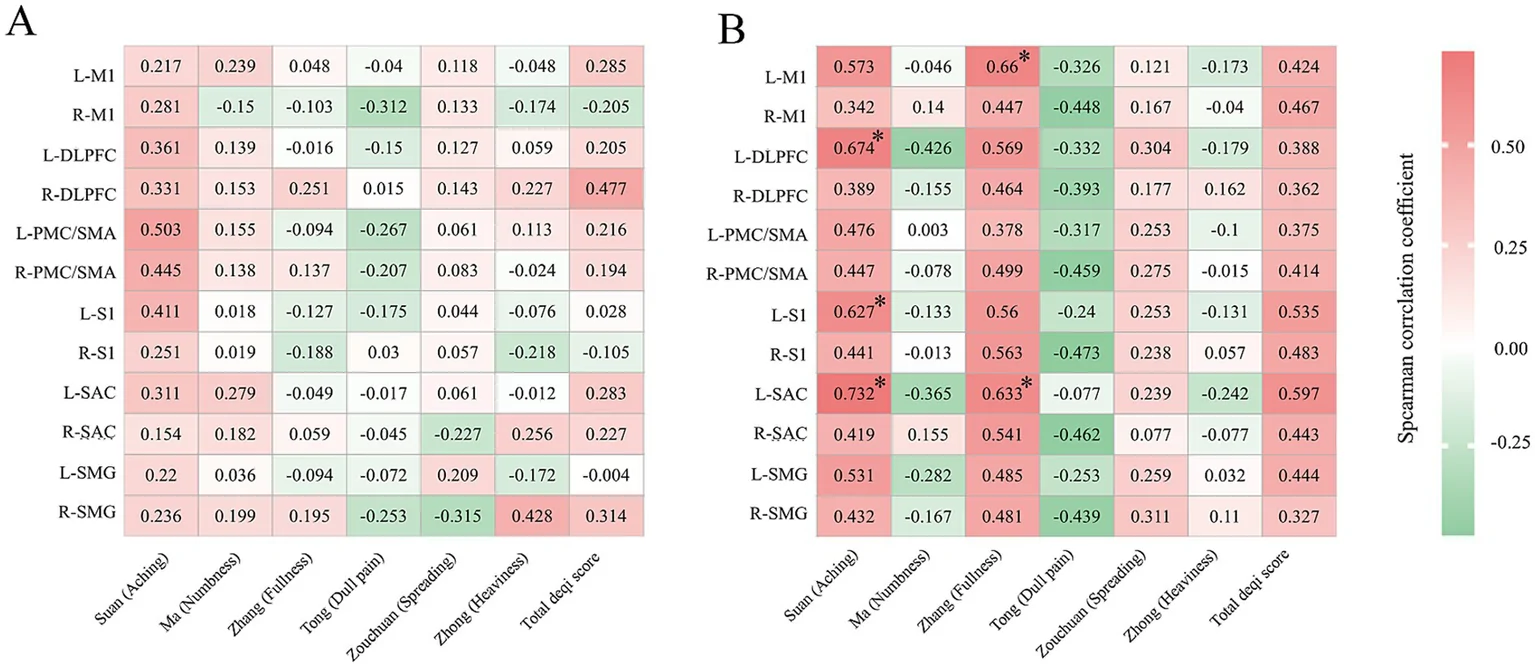
*Deqi* sensation–cortical activation correlations in the HC and PSD groups. **(A)** HC group. **(B)** PSD group. Color intensity and hue represent the Spearman correlation coefficient (*rho*), with red indicating positive correlations and green indicating negative correlations. All *p*-values were corrected using the FDR method. * *p* < 0.05.

No statistically significant correlations were observed in the HC group (*p* > 0.05) (Figure 5A). In the PSD group, activation in L-SAC was significantly positively correlated with both *suan* (aching) (*p* = 0.028, *rho* = 0.732, 95% CI [0.441, 0.886]) and *zhang* (fullness) (*p* = 0.034, *rho* = 0.633, 95% CI [0.280, 0.837]). Furthermore, *suan* (aching) was significantly positively correlated with activation in L-DLPFC (*p* = 0.028, *rho* = 0.674, 95% CI [0.343, 0.858]) and L-S1 (*p* = 0.034, *rho* = 0.627, 95% CI [0.271, 0.834]). *Zhang* (fullness) was also significantly positively correlated with L-M1 activation (*p* = 0.028, *rho* = 0.660, 95% CI [0.319, 0.851]) (Figure 5B). The full correlation matrix for both groups is presented in Supplementary Table 8.

## 4 Discussion

The clinical efficacy of acupuncture in improving PSD has been confirmed by meta-analysis (32); however, the neural mechanisms by which it modulates the central swallowing network remain unelucidated. Here, we used fNIRS under a standardized acupuncture paradigm at CV23 to compare cortical activation patterns and the association between *deqi* sensation and cortical activation between PSD patients and HC. The key findings are as follows. First, significant activation was observed in the contralesional sensorimotor network, including L-M1, L-S1, L-SAC, and L-SMG, in PSD patients, while only R-SAC showed significant activation in the ipsilesional hemisphere; no significant changes were observed in the HC group. Second, *suan* (aching) and *zhang* (fullness) sensations were positively correlated with activation in multiple brain regions of the contralesional hemisphere in the PSD group, whereas no significant FDR-corrected correlations were found in the HC group. These findings provide new insights into the association between acupuncture and the central mechanisms of PSD, which are discussed in detail below.

### 4.1 State-dependent relationship between *deqi* sensation and cortical activation

Intergroup comparison results demonstrated that during the needle manipulation period, HbO concentrations in L-M1, L-S1, L-SAC, and L-SMG were significantly higher in the PSD group than in the HC group. At the pre-stimulation baseline, no significant between-group differences in HbO or HbR concentrations were observed in any ROI, indicating that the two groups were hemodynamically comparable before acupuncture. The between-group HbO differences during the needle manipulation period therefore likely reflect acupuncture-evoked responses rather than pre-existing baseline differences. This predominantly contralesional activation pattern aligns with the theory of interhemispheric inhibition imbalance (33), which describes that following unilateral hemispheric stroke, excitability of the ipsilesional hemisphere decreases, while the contralesional hemisphere becomes hyperexcitable. Further, Yao et al. (34) confirmed via optogenetic experiments in a mouse model of PSD that the therapeutic effect of electroacupuncture at CV23 depended on specific activation of the contralesional M1. The enhanced activation of L-M1 observed in this study thus provides indirect human neuroimaging correlational evidence for this causal mechanism, suggesting that the contralesional M1 may be an important brain region involved in the central response to acupuncture.

Among these brain regions, L-S1, as the primary somatosensory cortex, is involved in sensory feedback processing and encoding during the oral phase of swallowing and serves as the main sensory driving input for swallowing regulation (35). L-SAC, located near the anterior parietal operculum, is responsible for cross-modal somatosensory information integration and interoceptive salience encoding (29). L-SMG constitutes the core of the parietal swallowing network, playing a central role in mediating oropharyngeal sensorimotor integration (28) and showing consistent activation during swallowing tasks (36). These three regions are, respectively, responsible for sensory encoding during the oral phase, cross-modal sensory integration, and oropharyngeal sensorimotor integration. Their synchronized activation with L-M1, which is involved in the motor control of swallowing, represents a characteristic acupuncture activation pattern of the contralesional hemisphere in individuals with stroke. Dai et al. (37) reported that electroacupuncture at CV23 could modulate contralesional cortical plasticity via the nucleus tractus solitarius (NTS)—ventral posteromedial nucleus (VPM) of the thalamus—S1 sensory afferent pathway, providing a potential neural circuit hypothesis for the activation of the contralesional hemisphere sensory cortex observed in our study. Notably, the significant HbO increase in L-SMG was accompanied by a significant HbR decrease, and L-S1 likewise showed a significant HbR decrease—a canonical neurovascular-coupling pattern that argues against a purely systemic vascular origin of the responses.

On the ipsilesional side, only R-SAC exhibited significant activation, whereas R-M1 and R-S1 showed no obvious response. The SAC serves as a higher-order center for somatosensory information integration, coordinating peripheral sensory afferents with motor planning (38). Given the significant activation of L-SAC observed in the present study, the ipsilesional SAC response may be attributable to transcallosal facilitation driven by the hyperactive contralesional hemisphere. As a higher-order association cortex, the SAC possesses dense interhemispheric connections via the corpus callosum, enabling the bilateral sharing of integrated sensory information (39). In contrast, the absence of significant activation in R-M1 and R-S1 may be attributed to impaired integrity of the corticospinal tract and thalamocortical tracts on the ipsilesional side (40), as these primary sensorimotor areas rely more heavily on direct thalamocortical inputs that are compromised by the stroke lesion.

No significant cortical activation was detected in the HC group following the same standardized acupuncture protocol. Combined with previous reports that acupuncture elicits distinct brain activation patterns in stroke patients versus healthy controls (41, 42), this finding preliminarily suggests that the cortical activation induced by acupuncture at CV23 may be pathologically state-dependent. Specifically, when the swallowing-related neural circuitry is impaired, cortical activation in the contralesional hemisphere is more pronounced, whereas no comparable changes are observed under physiological conditions. This phenomenon aligns with the theoretical framework of the state-dependent neuroimmune gateway (43), which posits that acupuncture primarily exerts its modulatory effects on sensitized neural circuits under pathological states while maintaining homeostatic stability in the healthy brain. Notably, our study did not include a sham acupuncture control, making it impossible to distinguish whether the observed cortical activation represents an acupuncture-specific response or a non-specific somatosensory stimulation effect. Nevertheless, this activation pattern may still provide a preliminary neuroimaging reference for the clinical development of cortical rehabilitation strategies in patients with PSD.

### 4.2 Associations between *deqi* sensation and cortical activation

Intergroup comparison of *deqi* scores demonstrated that PSD patients exhibited significantly higher total *deqi* scores, as well as higher scores for *suan* (aching) and *zhang* (fullness), than the HC group. No statistically significant differences were observed in other sensory dimensions. This finding is related to the afferent characteristics of acupuncture at CV23. With a needling depth of approximately 15 mm, the needle tip primarily contacts deep fascia and muscle layers, preferentially recruiting slow-adapting mechanoreceptors. The induced *deqi* sensation is therefore predominantly characterized by deep somatic sensations such as *suan* (aching) and *zhang* (fullness) (44). The mechanisms underlying the stronger subjective sensations in PSD patients in response to acupuncture stimulation of the same intensity may involve pathological disinhibition and sensory gain upregulation within the thalamocortical sensory circuit following stroke. Specifically, the imbalance in cross-hemispheric inhibition mediated by the thalamic reticular nucleus may lead to disinhibitory changes in the contralesional thalamocortical pathway, thereby amplifying cortical sensory signals (45, 46). Consequently, it is hypothesized that peripheral acupuncture afferent signals may be amplified in the contralesional cortex.

After FDR correction, five significant positive Spearman correlations were observed in the PSD group, all concentrated in the contralesional hemisphere, whereas no significant associations were found in the HC group. It should be noted that, in the HC group, the median scores for most *deqi* items were 0, with a markedly restricted score range. This floor effect mathematically limited the detectability of correlations, and the difference in correlation patterns between the two groups may be partly attributable to it.

Among the significant correlations in the PSD group, *zhang* (fullness) sensation was positively correlated with L-M1, and *suan* (aching) sensation was positively correlated with L-S1. During acupuncture at CV23, deep tissue signals are transmitted via the thalamus to the primary somatosensory cortex, producing a relatively diffuse *suan* (aching) sensation (47, 48). The perception of *zhang* (fullness) sensation additionally requires the involvement of the motor cortex (49); consistent with this, the correlation between L-M1 and *zhang* (fullness) sensation suggests that the contralesional motor cortex may be involved in sensorimotor integration (34). Furthermore, L-SAC was the only brain region significantly correlated with both *suan* (aching) and *zhang* (fullness) sensations. This dual correlation suggests that L-SAC, as a higher-order hub for multimodal sensory integration (50), may integrate *deqi*-related signals from different sensory dimensions and be involved in neural circuit activity within the contralesional swallowing network. Additionally, the significant positive correlation between L-DLPFC and *suan* (aching) sensation is consistent with the findings of Sun et al. (8), which demonstrated that acupuncture can enhance DLPFC network centrality. This observation suggests that the DLPFC may be one of the brain regions involved in the central response to acupuncture.

These findings, to some extent, echo the traditional Chinese medicine concept that *deqi* is essential for therapeutic efficacy (12). In patients with PSD, the more intense the core *deqi* sensations, the more pronounced the activation of contralesional target brain regions, whereas no such association was observed in the HC group. The underlying mechanism may involve functionally intact central inhibitory circuits in healthy individuals, which effectively gate somatosensory afferent signals through intracortical feedforward inhibition (51), thereby maintaining acupuncture-induced cortical responses at relatively low levels. Consequently, the correlation between *deqi* sensation and cortical activation fails to reach statistical significance after FDR correction. In contrast, stroke-induced damage may be accompanied by disinhibitory alterations in the thalamocortical circuit (45, 46), disrupting homeostatic regulation and thereby markedly amplifying the cortical transmission of acupuncture signals. The observed association pattern suggests that the correlation strength between *deqi* sensation and cortical activation may carry reference value, providing a basis for individualized acupuncture regimens and appropriate stimulation intensity in clinical practice. However, given that this correlation analysis did not include an *a priori* power analysis and was constrained by sample size, the correlation results should be regarded as exploratory findings. Their robustness warrants further validation in larger samples.

### 4.3 Limitations

This study has some limitations. First, this was a single-center cross-sectional study observing only the immediate cortical activation effects induced by a single acupuncture session, with no longitudinal clinical outcome data collected; thus, a causal relationship between acupuncture-evoked cortical responses and long-term improvements in swallowing function cannot be established. In addition, the *deqi* VAS was completed once after needle withdrawal to cover the three needle manipulation blocks, which may have introduced recall bias. Secondly, although an *a priori* power analysis was conducted, the sample size of the present study remains relatively small, and between-group comparisons were not based on baseline-corrected change values, which may compromise the statistical robustness and generalizability of the results. Furthermore, the correlation analyses were not preceded by an *a priori* power analysis, and the corresponding results should therefore be regarded as exploratory findings. Third, only PSD patients with unilateral supratentorial lesions were enrolled. Moreover, although the ipsilesional side was spatially standardized in the group-level analysis via mirror flipping, inter-individual differences in lateralization cannot be completely excluded, which limits the generalizability of the current findings to the broader PSD population. Fourth, no sham acupuncture control condition was included in this study, making it impossible to distinguish whether the observed cortical activation represents an acupoint-specific response or is elicited by non-specific somatosensory stimulation; accordingly, the acupoint specificity of cortical activation cannot be definitively determined. Fifth, the fNIRS device used in this study was not equipped with short-separation channels, precluding correction of superficial signals. With its limited spatial resolution, fNIRS can only detect cortical hemodynamic changes and cannot capture neural activity in subcortical structures such as the brainstem and thalamus. Moreover, the single-channel ROIs were susceptible to local coupling quality, hair interference, and partial volume effects. Collectively, these factors limited the depth of interpretation of acupuncture-related cortical hemodynamic responses and the analysis of their association with the entire swallowing neural network.

## 5 Conclusion

Acupuncture at CV23 is associated with altered cortical activation in the contralesional sensorimotor network, including L-M1, L-S1, L-SAC, and L-SMG, in patients with PSD, whereas no comparable cortical responses were detected in healthy controls. *Suan* (aching) and *zhang* (fullness) sensations were significantly positively correlated with multi-region activation in the contralesional hemisphere in PSD patients, whereas no significant FDR-corrected correlations were found in the healthy controls. These findings provide fNIRS neuroimaging evidence for understanding the association between acupuncture and the central mechanisms of PSD, and offer a valuable reference for exploring individualized cortex-targeted acupuncture rehabilitation protocols. Future large-sample longitudinal studies incorporating sham acupuncture controls and multimodal imaging techniques are warranted to further validate the present findings.

## Data Availability

The original contributions presented in the study are included in the article/Supplementary material, further inquiries can be directed to the corresponding authors.
