# Supplementary material for: Cortical hemodynamic responses to acupuncture at Lianquan (CV23) in patients with post-stroke dysphagia: a functional near-infrared spectroscopy study
Source: Front Neurol. 2026 Sep 9;17:1921352. doi: 10.3389/fneur.2026.1921352 (PMC13597691; doi:10.3389/fneur.2026.1921352)
Supplement: Supplementary file 1 [file Table_1.docx]

Supplementary Material

Supplementary Tables

Supplementary Table 1. Visual Analog Scale (VAS) for *deqi* sensation assessment.

| Item | Original term | English description | Scale range |
| --- | --- | --- | --- |
| *suan* | 酸 (Suān) | Aching or soreness | 0-10 |
| *ma* | 麻 (Má) | Numbness or tingling | 0-10 |
| *zhang* | 胀 (Zhàng) | Fullness, distention, or pressure | 0-10 |
| *tong* | 痛 (Tòng) | Dull pain | 0-10 |
| *zouchuan* | 走窜 (Zǒuchuàn) | Spreading sensation from the needling site to surrounding areas | 0-10 |
| *zhong* | 重 (Zhòng) | Heaviness | 0-10 |

Note: VAS, Visual Analog Scale. Each item is rated on a continuous scale from 0 (no sensation) to 10 (unbearable intense sensation). The total *deqi* score is the sum of all six item scores, ranging from 0 to 60. This VAS instrument was adapted from previous acupuncture *deqi* sensation studies (17).

Supplementary Table 2. Signal quality of fNIRS channels assessed by the coefficient of variation (CV) at 730 nm and 850 nm.

| ROI | Ch | PSD group (*n*=21) | | | | |  | HC group (*n*=29) | | | | |
| --- | --- | --- | --- | --- | --- | --- | --- | --- | --- | --- | --- | --- |
|  |  | 730 nm mean (%) | 730 nm range (%) | 850 nm mean (%) | 850 nm range (%) | Ch excluded |  | 730 nm mean (%) | 730 nm range (%) | 850 nm mean (%) | 850 nm range (%) | Ch excluded |
| L-M1 | 32 | 3.46 | 0.93–11.91 | 3.67 | 0.96–10.10 | 0 |  | 4.10 | 1.59–10.19 | 4.22 | 1.36–10.06 | 0 |
| R-M1 | 13 | 3.21 | 0.83–8.94 | 3.31 | 1.02–7.31 | 0 |  | 5.01 | 1.30–13.78 | 5.17 | 1.84–13.39 | 0 |
| L-PMC/SMA | 28 | 1.93 | 0.64–5.27 | 2.45 | 1.04–5.76 | 0 |  | 3.23 | 0.84–10.99 | 3.63 | 0.74–11.25 | 0 |
|  | 33 | 2.12 | 0.69–5.25 | 2.49 | 1.09–5.19 | 0 |  | 3.60 | 1.05–10.86 | 4.04 | 1.67–10.32 | 1 |
|  | 34 | 1.83 | 0.52–5.07 | 2.26 | 0.85–5.04 | 0 |  | 2.80 | 0.71–9.15 | 3.52 | 1.11–11.17 | 0 |
|  | 41 | 3.02 | 0.60–10.39 | 3.21 | 0.97–9.05 | 0 |  | 3.43 | 0.90–9.57 | 4.08 | 1.04–14.58 | 0 |
|  | 43 | 2.60 | 0.67–8.32 | 2.80 | 1.05–8.14 | 0 |  | 3.49 | 1.12–8.19 | 4.05 | 1.59–13.78 | 0 |
|  | 49 | 3.27 | 1.15–11.20 | 3.38 | 1.55–10.01 | 1 |  | 3.72 | 1.11–8.49 | 4.01 | 1.39–10.52 | 0 |
| R-PMC/SMA | 2 | 3.17 | 0.89–13.07 | 3.30 | 1.07–13.59 | 0 |  | 3.63 | 0.58–9.70 | 4.03 | 0.84–8.51 | 0 |
|  | 6 | 2.24 | 0.62–11.25 | 2.56 | 1.05–11.45 | 0 |  | 4.11 | 1.11–14.20 | 4.70 | 1.04–13.47 | 1 |
|  | 8 | 2.39 | 0.48–6.63 | 2.85 | 1.05–6.70 | 0 |  | 4.40 | 1.39–13.54 | 4.84 | 1.46–12.99 | 0 |
|  | 14 | 3.04 | 0.66–9.81 | 3.32 | 1.14–9.26 | 0 |  | 4.12 | 1.23–14.48 | 4.38 | 1.13–13.50 | 1 |
|  | 15 | 2.23 | 0.60–8.92 | 2.55 | 0.67–9.18 | 1 |  | 2.70 | 0.92–9.10 | 3.63 | 1.24–11.01 | 1 |
|  | 17 | 2.92 | 0.40–10.42 | 3.16 | 0.68–10.50 | 0 |  | 4.05 | 0.80–14.47 | 3.92 | 1.38–11.44 | 0 |
| L-DLPFC | 35 | 1.77 | 0.66–3.51 | 2.21 | 0.94–4.83 | 0 |  | 3.24 | 1.18–11.73 | 3.81 | 1.18–13.23 | 0 |
|  | 36 | 2.49 | 0.85–11.68 | 3.51 | 1.42–13.09 | 0 |  | 2.73 | 0.83–6.87 | 3.44 | 0.92–9.65 | 1 |
|  | 44 | 1.54 | 0.61–3.56 | 2.25 | 0.95–4.06 | 0 |  | 2.68 | 0.88–9.15 | 3.35 | 1.37–13.19 | 1 |
| R-DLPFC | 9 | 2.83 | 0.69–14.65 | 2.85 | 0.85–13.95 | 0 |  | 3.02 | 1.25–10.11 | 4.16 | 1.27–14.23 | 1 |
|  | 10 | 2.07 | 0.50–5.37 | 2.41 | 0.67–8.02 | 0 |  | 2.94 | 1.18–11.82 | 3.42 | 1.08–11.27 | 1 |
|  | 19 | 2.32 | 0.64–7.09 | 2.91 | 0.96–6.36 | 1 |  | 3.02 | 0.82–10.60 | 3.84 | 1.49–10.70 | 1 |
|  | 20 | 1.66 | 0.72–3.20 | 2.18 | 1.32–4.82 | 1 |  | 3.07 | 0.60–11.49 | 3.90 | 1.16–10.17 | 0 |
| L-SMG | 39 | 2.68 | 0.92–6.51 | 2.82 | 1.22–4.48 | 1 |  | 3.93 | 1.08–11.40 | 4.23 | 1.07–14.28 | 0 |
|  | 48 | 2.61 | 1.25–10.12 | 2.82 | 1.28–9.89 | 0 |  | 3.56 | 1.03–13.79 | 3.54 | 1.17–13.60 | 0 |
| R-SMG | 1 | 2.86 | 0.88–10.07 | 3.23 | 1.07–10.83 | 0 |  | 3.32 | 1.10–9.96 | 3.85 | 1.49–9.56 | 0 |
|  | 3 | 2.50 | 0.89–6.68 | 2.76 | 1.04–6.25 | 0 |  | 4.24 | 0.99–13.94 | 5.09 | 0.64–13.99 | 1 |
| L-S1 | 27 | 2.86 | 0.91–11.95 | 2.95 | 1.08–12.07 | 0 |  | 4.35 | 1.77–12.61 | 4.01 | 1.67–11.24 | 0 |
|  | 40 | 3.54 | 0.92–11.83 | 3.59 | 0.94–12.44 | 0 |  | 3.44 | 1.13–8.38 | 4.17 | 1.45–14.82 | 0 |
|  | 42 | 2.79 | 1.20–7.10 | 2.97 | 1.47–8.01 | 0 |  | 3.33 | 1.15–8.54 | 3.65 | 1.11–12.37 | 1 |
| R-S1 | 5 | 2.90 | 1.32–6.40 | 2.99 | 1.59–6.87 | 0 |  | 4.29 | 1.02–11.29 | 5.03 | 1.64–12.30 | 1 |
|  | 7 | 3.80 | 0.46–12.34 | 3.92 | 0.74–9.26 | 0 |  | 4.46 | 1.50–12.74 | 4.75 | 1.74–13.28 | 1 |
|  | 16 | 2.98 | 0.98–8.06 | 3.03 | 1.07–7.10 | 0 |  | 4.19 | 0.92–10.01 | 4.13 | 1.07–8.77 | 0 |
| L-SAC | 38 | 2.99 | 1.10–9.43 | 3.37 | 1.07–9.73 | 0 |  | 3.96 | 1.12–12.02 | 4.47 | 1.45–12.69 | 0 |
| R-SAC | 4 | 2.56 | 0.86–8.51 | 2.92 | 1.21–9.02 | 0 |  | 4.80 | 0.79–13.28 | 5.14 | 1.08–14.77 | 0 |

Note: CV, coefficient of variation. CV was calculated as (standard deviation/mean) × 100 based on the raw optical intensity at each wavelength. Channels with a CV ≥ 15% at either wavelength in any participant were excluded from further analysis; the number of excluded subject–channel combinations is reported for each channel. Ch, channel number. Ranges are the minimum–maximum CV values across participants. ROI abbreviations are defined in Table 1.

Supplementary Table 3. Within-group comparisons of HbO concentration changes between the pre-stimulation baseline and needle manipulation period.

| ROI | HC group (*n* = 29) | | | | PSD group (*n* = 21) | | | |
| --- | --- | --- | --- | --- | --- | --- | --- | --- |
|  | Baseline | Manipulation | *p* | Effect size [95% CI] | Baseline | Manipulation | *p* | Effect size [95% CI] |
| L-M1 | 0.000 ± 0.044 | -0.025 ± 0.053 | 0.288 | d = 0.286 [–0.088, 0.655] | -0.021 ± 0.025 | 0.014 ± 0.037 | 0.040* | d = 0.646 [0.168, 1.112] |
| R-M1† | -0.003 (-0.039, 0.037) | -0.004 (-0.038, 0.035) | 0.957 | r = 0.010 [–0.357, 0.374] | -0.021 (-0.028, 0.004) | 0.016 (-0.020, 0.033) | 0.108 | r = 0.421 [–0.013, 0.721] |
| L-PMC/SMA | 0.002 ± 0.036 | -0.014 ± 0.042 | 0.324 | d = 0.221 [–0.149, 0.588] | -0.008 ± 0.034 | 0.009 ± 0.030 | 0.277 | d = 0.284 [–0.156, 0.717] |
| R-PMC/SMA | 0.002 ± 0.032 | -0.013 ± 0.033 | 0.288 | d = 0.265 [–0.108, 0.633] | -0.009 ± 0.027 | 0.008 ± 0.036 | 0.277 | d = 0.287 [–0.153, 0.720] |
| L-DLPFC | 0.000 ± 0.029 | -0.013 ± 0.045 | 0.355 | d = 0.198 [–0.171, 0.564] | -0.008 ± 0.033 | 0.005 ± 0.034 | 0.346 | d = 0.224 [–0.212, 0.655] |
| R-DLPFC | 0.003 ± 0.034 | -0.013 ± 0.041 | 0.288 | d = 0.263 [–0.110, 0.631] | -0.008 ± 0.030 | 0.005 ± 0.035 | 0.346 | d = 0.226 [–0.210, 0.657] |
| L-SMG | 0.001 ± 0.037 | -0.017 ± 0.047 | 0.288 | d = 0.272 [–0.101, 0.641] | -0.015 ± 0.023 | 0.014 ± 0.033 | 0.040* | d = 0.651 [0.172, 1.117] |
| R-SMG† | 0.008 (-0.034, 0.034) | 0.002 (-0.038, 0.020) | 0.391 | r = 0.171 [–0.208, 0.504] | -0.005 ± 0.029 | 0.006 ± 0.033 | 0.366 | d = 0.202 [–0.233, 0.632] |
| L-S1 | 0.005 ± 0.049 | -0.024 ± 0.048 | 0.288 | d = 0.310 [–0.060, 0.676] | -0.012 ± 0.021 | 0.012 ± 0.034 | 0.084 | d = 0.492 [0.033, 0.941] |
| R-S1† | 0.007 ± 0.049 | -0.016 ± 0.051 | 0.311 | d = 0.240 [–0.131, 0.607] | -0.011 (-0.023, 0.012) | 0.004 (-0.010, 0.038) | 0.209 | r = 0.338 [–0.110, 0.670] |
| L-SAC | 0.006 ± 0.053 | -0.027 ± 0.051 | 0.288 | d = 0.349 [–0.029, 0.721] | -0.017 ± 0.040 | 0.023 ± 0.034 | 0.040* | d = 0.617 [0.143, 1.079] |
| R-SAC† | 0.008 ± 0.044 | -0.026 ± 0.055 | 0.288 | d = 0.378 [–0.002, 0.752] | -0.016 (-0.026, 0.003) | 0.015 (-0.007, 0.034) | 0.084 | r = 0.474 [0.053, 0.752] |

Note: Normally distributed data are presented as mean ± SD and compared using paired-samples t-test, with Cohen's d as the effect size and its 95% CI calculated based on the noncentral t-distribution. † Non-normally distributed data are presented as median (P₂₅, P₇₅) and compared using Wilcoxon signed-rank test, with the rank-biserial correlation coefficient *r* as the effect size and its 95% CI approximated using Fisher’s z transformation. All *p*-values were corrected using the FDR method. ROI abbreviations are defined in Table 1. **p* < 0.05.

Supplementary Table 4. Within-group comparisons of deoxygenated hemoglobin (HbR) concentration changes between the pre-stimulation baseline and needle manipulation period.

| ROI | HC group (*n* = 29) | | | | PSD group (*n* = 21) | | | |
| --- | --- | --- | --- | --- | --- | --- | --- | --- |
|  | Baseline | Manipulation | *p* | Effect size [95% CI] | Baseline | Manipulation | *p* | Effect size [95% CI] |
| L-M1 | 0.001 ± 0.039 | 0.004 ± 0.044 | 0.889 | *d* = –0.026 [–0.390, 0.338] | 0.005 ± 0.013 | –0.005 ± 0.013 | 0.080 | *d* = 0.552 [0.085, 1.006] |
| R-M1 | 0.017 ± 0.045 | –0.012 ± 0.046 | 0.328 | *d* = 0.334 [–0.043, 0.706] | –0.002 ± 0.020 | –0.002 ± 0.015 | 0.969 | *d* = –0.009 [–0.436, 0.419] |
| L-DLPFC | 0.000 ± 0.020 | 0.001 ± 0.018 | 0.889 | *d* = –0.032 [–0.395, 0.333] | 0.003 ± 0.014 | –0.000 ± 0.013 | 0.657 | *d* = 0.166 [–0.267, 0.595] |
| R-DLPFC | –0.000 ± 0.020 | –0.002 ± 0.022 | 0.889 | *d* = 0.047 [–0.318, 0.410] | 0.001 ± 0.015 | 0.000 ± 0.013 | 0.856 | *d* = 0.060 [–0.369, 0.488] |
| L-PMC/SMA† | 0.006 (–0.008, 0.019) | –0.005 (–0.022, 0.012) | 0.825 | *r* = 0.203 [–0.177, 0.530] | 0.002 ± 0.014 | –0.000 ± 0.015 | 0.840 | *d* = 0.085 [–0.344, 0.513] |
| R-PMC/SMA | 0.002 ± 0.022 | –0.003 ± 0.022 | 0.889 | *d* = 0.125 [–0.242, 0.489] | –0.002 ± 0.013 | 0.003 ± 0.014 | 0.657 | *d* = –0.170 [–0.599, 0.263] |
| L-S1† | 0.004 ± 0.029 | 0.006 ± 0.027 | 0.889 | *d* = –0.041 [–0.404, 0.324] | 0.005 (–0.001, 0.012) | –0.001 (–0.014, 0.004) | 0.042* | *r* = 0.588 [0.209, 0.813] |
| R-S1 | 0.013 ± 0.035 | –0.012 ± 0.030 | 0.234 | *d* = 0.418 [0.035, 0.795] | –0.002 ± 0.019 | 0.003 ± 0.021 | 0.657 | *d* = –0.152 [–0.581, 0.280] |
| L-SAC | –0.002 ± 0.043 | 0.005 ± 0.040 | 0.889 | *d* = –0.083 [–0.447, 0.282] | 0.013 ± 0.022 | 0.003 ± 0.015 | 0.096 | *d* = 0.502 [0.042, 0.951] |
| R-SAC | 0.006 ± 0.055 | –0.002 ± 0.045 | 0.889 | *d* = 0.083 [–0.283, 0.447] | –0.002 ± 0.016 | 0.006 ± 0.019 | 0.475 | *d* = –0.291 [–0.724, 0.150] |
| L-SMG† | 0.007 (–0.012, 0.017) | –0.003 (–0.012, 0.010) | 0.889 | *r* = 0.106 [–0.271, 0.455] | 0.005 (–0.002, 0.008) | –0.002 (–0.017, 0.002) | 0.024* | *r* = 0.671 [0.337, 0.855] |
| R-SMG† | 0.006 (–0.008, 0.033) | –0.013 (–0.032, 0.007) | 0.234 | *r* = 0.383 [0.020, 0.658] | –0.003 ± 0.016 | 0.004 ± 0.017 | 0.594 | *d* = –0.233 [–0.664, 0.203] |

Note: Normally distributed data are presented as mean ± SD and compared using paired-samples t-test, with Cohen's d as the effect size and its 95% CI calculated based on the noncentral t-distribution. † Non-normally distributed data are presented as median (P₂₅, P₇₅) and compared using Wilcoxon signed-rank test, with the rank-biserial correlation coefficient r as the effect size and its 95% CI approximated using Fisher's z transformation. All *p*-values were corrected using the FDR method. ROI abbreviations are defined in Table 1. **p* < 0.05.

Supplementary Table 5. Between-group comparisons of HbO concentration during the pre-stimulation baseline period.

| ROI | HC group  (*n* = 29) | PSD group  (*n* = 21) | *p* | Effect size [95% CI] |
| --- | --- | --- | --- | --- |
| L-M1† | 0.004 (–0.030, 0.020) | –0.016 (–0.032, –0.003) | 0.281 | *r* = 0.263 [–0.014, 0.509] |
| R-M1† | –0.003 (–0.039, 0.037) | –0.021 (–0.028, 0.004) | 0.331 | *r* = 0.138 [–0.137, 0.396] |
| L-PMC/SMA | 0.002 ± 0.036 | –0.008 ± 0.034 | 0.331 | *d* = –0.282 [–0.845, 0.284] |
| R-PMC/SMA† | –0.001 (–0.019, 0.031) | –0.014 (–0.019, 0.000) | 0.281 | *r* = 0.179 [–0.095, 0.431] |
| L-DLPFC† | –0.002 (–0.020, 0.027) | –0.011 (–0.024, 0.009) | 0.281 | *r* = 0.168 [–0.107, 0.422] |
| R-DLPFC | 0.003 ± 0.034 | –0.008 ± 0.030 | 0.281 | *d* = –0.351 [–0.915, 0.217] |
| L-SMG | 0.001 ± 0.037 | –0.015 ± 0.023 | 0.281 | *d* = –0.487 [–1.055, 0.085] |
| R-SMG† | 0.008 (–0.034, 0.034) | –0.008 (–0.028, 0.008) | 0.281 | *r* = 0.188 [–0.088, 0.439] |
| L-S1 | 0.005 ± 0.049 | –0.012 ± 0.021 | 0.281 | *d* = –0.419 [–0.985, 0.151] |
| R-S1† | 0.003 (–0.018, 0.037) | –0.011 (–0.023, 0.012) | 0.281 | *r* = 0.196 [–0.079, 0.445] |
| L-SAC | 0.006 ± 0.053 | –0.017 ± 0.040 | 0.281 | *d* = –0.493 [–1.061, 0.079] |
| R-SAC† | –0.000 (–0.015, 0.048) | –0.016 (–0.026, 0.003) | 0.281 | *r* = 0.179 [–0.095, 0.431] |

Note: Normally distributed data are presented as mean ± SD and compared using independent samples t-test (Welch's t-test was applied when the assumption of equal variances was violated), with Cohen's d as the effect size and its 95% CI calculated based on the noncentral t-distribution. † Non-normally distributed data are presented as median (P₂₅, P₇₅) and compared using Mann–Whitney U test, with the rank-biserial correlation coefficient r as the effect size and its 95% CI approximated using Fisher's z transformation. All *p*-values were corrected using the FDR method. ROI abbreviations are defined in Table 1.

Supplementary Table 6. Between-group comparisons of deoxygenated hemoglobin (HbR) concentration during the pre-stimulation baseline period and the needle manipulation period.

| ROI | Pre-stimulation baseline period | | | |  | ROI | Needle manipulation period | | | |
| --- | --- | --- | --- | --- | --- | --- | --- | --- | --- | --- |
|  | HC group | PSD group | p | Effect size [95% CI] |  |  | HC group | PSD group | p | Effect size [95% CI] |
| L-M1 | 0.001 ± 0.039 | 0.005 ± 0.013 | 0.791 | *d* = 0.108 [–0.455, 0.669] |  | L-M1† | 0.010 (–0.019, 0.020) | –0.002 (–0.014, 0.003) | 0.327 | *r* = 0.227 [–0.055, 0.475] |
| R-M1 | 0.017 ± 0.045 | –0.002 ± 0.020 | 0.262 | *d* = –0.531 [–1.100, 0.043] |  | R-M1 | –0.012 ± 0.046 | –0.002 ± 0.015 | 0.569 | *d* = 0.273 [–0.292, 0.836] |
| L-PMC/SMA† | 0.006 (–0.008, 0.019) | 0.003 (–0.007, 0.011) | 0.784 | *r* = 0.090 [–0.193, 0.360] |  | L-PMC/SMA† | –0.005 (–0.022, 0.012) | 0.002 (–0.008, 0.011) | 0.767 | *r* = 0.079 [–0.204, 0.350] |
| R-PMC/SMA† | 0.010 (–0.007, 0.018) | –0.002 (–0.010, 0.006) | 0.262 | *r* = 0.238 [–0.044, 0.484] |  | R-PMC/SMA | –0.003 ± 0.022 | 0.003 ± 0.014 | 0.569 | *d* = 0.281 [–0.285, 0.844] |
| L-DLPFC | 0.000 ± 0.020 | 0.003 ± 0.014 | 0.784 | *d* = 0.185 [–0.379, 0.746] |  | L-DLPFC | 0.001 ± 0.018 | –0.000 ± 0.013 | 0.847 | *d* = –0.082 [–0.643, 0.480] |
| R-DLPFC | –0.000 ± 0.020 | 0.001 ± 0.015 | 0.791 | *d* = 0.101 [–0.461, 0.663] |  | R-DLPFC | –0.002 ± 0.022 | 0.000 ± 0.013 | 0.767 | *d* = 0.125 [–0.438, 0.686] |
| L-SMG† | 0.007 (–0.012, 0.017) | 0.005 (–0.002, 0.008) | 0.785 | *r* = 0.077 [–0.207, 0.348] |  | L-SMG† | –0.003 (–0.012, 0.010) | –0.002 (–0.017, 0.002) | 0.569 | *r* = 0.149 [–0.135, 0.411] |
| R-SMG† | 0.006 (–0.008, 0.033) | –0.002 (–0.012, 0.008) | 0.262 | *r* = 0.227 [–0.055, 0.475] |  | R-SMG† | –0.013 (–0.032, 0.007) | 0.008 (–0.008, 0.013) | 0.168 | *r* = 0.341 [0.069, 0.566] |
| L-S1 | 0.004 ± 0.029 | 0.005 ± 0.009 | 0.829 | *d* = 0.055 [–0.507, 0.616] |  | L-S1† | 0.005 (–0.010, 0.024) | –0.001 (–0.014, 0.004) | 0.168 | *r* = 0.310 [0.035, 0.542] |
| R-S1 | 0.013 ± 0.035 | –0.002 ± 0.019 | 0.262 | *d* = –0.509 [–1.077, 0.065] |  | R-S1 | –0.012 ± 0.030 | 0.003 ± 0.021 | 0.184 | *d* = 0.586 [0.010, 1.157] |
| L-SAC | –0.002 ± 0.043 | 0.013 ± 0.022 | 0.262 | *d* = 0.429 [–0.141, 0.995] |  | L-SAC | 0.005 ± 0.040 | 0.003 ± 0.015 | 0.853 | *d* = –0.047 [–0.609, 0.515] |
| R-SAC† | 0.006 (–0.010, 0.029) | –0.002 (–0.008, 0.003) | 0.266 | *r* = 0.213 [–0.070, 0.464] |  | R-SAC | –0.002 ± 0.045 | 0.006 ± 0.019 | 0.632 | *d* = 0.208 [–0.356, 0.770] |

Note: Normally distributed data are presented as mean ± SD and compared using independent samples t-test (Welch's t-test was applied when the assumption of equal variances was violated), with Cohen's d as the effect size and its 95% CI calculated based on the noncentral t-distribution. † Non-normally distributed data are presented as median (P₂₅, P₇₅) and compared using Mann–Whitney U test, with the rank-biserial correlation coefficient r as the effect size and its 95% CI approximated using Fisher's z transformation. All *p*-values were corrected using the FDR method. ROI abbreviations are defined in Table 1.

Supplementary Table 7. Between-group comparisons of HbO concentration during the needle manipulation period.

| ROI | HC group (*n* = 29) | PSD group (*n* = 21) | *p* | Effect size [95% CI] |
| --- | --- | --- | --- | --- |
| L-M1 | –0.025 ± 0.053 | 0.014 ± 0.037 | 0.014* | *d* = 0.834 [0.244, 1.415] |
| R-M1† | –0.004 (–0.038, 0.035) | 0.016 (–0.020, 0.033) | 0.415 | *r* = 0.115 [–0.170, 0.381] |
| L-PMC/SMA | –0.014 ± 0.042 | 0.009 ± 0.030 | 0.067 | *d* = 0.609 [0.032, 1.181] |
| R-PMC/SMA | –0.013 ± 0.033 | 0.008 ± 0.036 | 0.066 | *d* = 0.629 [0.051, 1.201] |
| L-DLPFC | –0.013 ± 0.045 | 0.005 ± 0.034 | 0.163 | *d* = 0.435 [–0.136, 1.001] |
| R-DLPFC | –0.013 ± 0.041 | 0.005 ± 0.035 | 0.143 | *d* = 0.470 [–0.102, 1.037] |
| L-SMG† | –0.008 (–0.029, 0.010) | 0.021 (–0.006, 0.039) | 0.014* | *r* = 0.388 [0.122, 0.602] |
| R-SMG | –0.009 ± 0.043 | 0.006 ± 0.033 | 0.213 | *d* = 0.377 [–0.192, 0.942] |
| L-S1 | –0.024 ± 0.048 | 0.012 ± 0.034 | 0.014* | *d* = 0.847 [0.256, 1.429] |
| R-S1† | –0.010 (–0.050, 0.023) | 0.004 (–0.010, 0.038) | 0.123 | *r* = 0.246 [–0.036, 0.490] |
| L-SAC | –0.027 ± 0.051 | 0.023 ± 0.034 | 0.012* | *d* = 1.097 [0.489, 1.694] |
| R-SAC | –0.026 ± 0.055 | 0.015 ± 0.036 | 0.014* | *d* = 0.854 [0.263, 1.437] |

Note: Normally distributed data are presented as mean ± SD and compared using independent samples t-test (Welch's t-test was applied when the assumption of equal variances was violated), with Cohen's d as the effect size and its 95% CI calculated based on the noncentral t-distribution. † Non-normally distributed data are presented as median (P₂₅, P₇₅) and compared using Mann-Whitney U test, with the rank-biserial correlation coefficient *r* as the effect size and its 95% CI approximated using Fisher’s z transformation. All *p-*values were corrected using the FDR method. ROI abbreviations are defined in Table 1. **p* < 0.05.

Supplementary Table 8. Full correlation matrix between *deqi* sensations and cortical activation during manipulation in PSD and HC groups.

| Brain region | *Deqi* item | HC *rho* [95% CI] | HC *p* | PSD *rho* [95% CI] | PSD *p* |
| --- | --- | --- | --- | --- | --- |
| L-M1 | *suan* (aching) | 0.217 [–0.162, 0.540] | 0.730 | 0.573 [0.187, 0.805] | 0.084 |
| L-M1 | *ma* (numbness) | 0.239 [–0.139, 0.558] | 0.730 | –0.046 [–0.468, 0.394] | 0.895 |
| L-M1 | *zhang* (fullness) | 0.048 [–0.328, 0.413] | 0.959 | 0.660 [0.319, 0.851] | 0.028* |
| L-M1 | *tong* (dull pain) | –0.040 [–0.405, 0.335] | 0.962 | –0.326 [–0.664, 0.123] | 0.272 |
| L-M1 | *zouchuan* (spreading) | 0.118 [–0.261, 0.465] | 0.843 | 0.121 [–0.325, 0.521] | 0.713 |
| L-M1 | *zhong* (heaviness) | –0.048 [–0.412, 0.328] | 0.959 | –0.173 [–0.557, 0.275] | 0.614 |
| L-M1 | Total *deqi* score | 0.285 [–0.091, 0.589] | 0.730 | 0.424 [–0.009, 0.723] | 0.140 |
| R-M1 | *suan* (aching) | 0.281 [–0.095, 0.586] | 0.730 | 0.342 [–0.103, 0.678] | 0.252 |
| R-M1 | *ma* (numbness) | –0.150 [–0.487, 0.227] | 0.802 | 0.140 [–0.307, 0.535] | 0.672 |
| R-M1 | *zhang* (fullness) | –0.103 [–0.450, 0.274] | 0.875 | 0.447 [0.018, 0.738] | 0.132 |
| R-M1 | *tong* (dull pain) | –0.312 [–0.605, 0.059] | 0.730 | –0.448 [–0.739, –0.019] | 0.132 |
| R-M1 | *zouchuan* (spreading) | –0.174 [–0.504, 0.206] | 0.807 | –0.040 [–0.463, 0.398] | 0.617 |
| R-M1 | *zhong* (heaviness) | 0.133 [–0.247, 0.477] | 0.760 | 0.167 [–0.282, 0.555] | 0.905 |
| R-M1 | Total *deqi* score | –0.205 [–0.527, 0.173] | 0.730 | 0.467 [0.041, 0.750] | 0.131 |
| L-PMC/SMA | *suan* (aching) | 0.503 [0.172, 0.730] | 0.378 | 0.476 [0.052, 0.756] | 0.131 |
| L-PMC/SMA | *ma* (numbness) | 0.155 [–0.222, 0.491] | 0.802 | –0.426 [–0.725, –0.003] | 0.140 |
| L-PMC/SMA | *zhang* (fullness) | –0.094 [–0.443, 0.282] | 0.895 | 0.569 [0.179, 0.809] | 0.084 |
| L-PMC/SMA | *tong* (dull pain) | –0.267 [–0.572, 0.108] | 0.730 | –0.332 [–0.668, 0.116] | 0.269 |
| L-PMC/SMA | *zouchuan* (spreading) | 0.061 [–0.315, 0.418] | 0.959 | 0.304 [–0.147, 0.654] | 0.309 |
| L-PMC/SMA | *zhong* (heaviness) | 0.113 [–0.266, 0.461] | 0.852 | –0.179 [–0.561, 0.269] | 0.609 |
| L-PMC/SMA | Total *deqi* score | 0.216 [–0.163, 0.539] | 0.730 | 0.388 [–0.047, 0.706] | 0.181 |
| R-PMC/SMA | *suan* (aching) | 0.445 [0.097, 0.696] | 0.441 | 0.447 [0.018, 0.738] | 0.132 |
| R-PMC/SMA | *ma* (numbness) | 0.138 [–0.240, 0.480] | 0.803 | –0.078 [–0.494, 0.365] | 0.807 |
| R-PMC/SMA | *zhang* (fullness) | 0.137 [–0.241, 0.479] | 0.803 | 0.499 [0.085, 0.770] | 0.126 |
| R-PMC/SMA | *tong* (dull pain) | –0.207 [–0.529, 0.171] | 0.730 | –0.459 [–0.746, –0.034] | 0.131 |
| R-PMC/SMA | *zouchuan* (spreading) | 0.083 [–0.295, 0.436] | 0.934 | 0.275 [–0.179, 0.636] | 0.374 |
| R-PMC/SMA | *zhong* (heaviness) | –0.024 [–0.391, 0.350] | 0.962 | –0.015 [–0.443, 0.419] | 0.965 |
| R-PMC/SMA | Total *deqi* score | 0.194 [–0.186, 0.522] | 0.730 | 0.414 [–0.021, 0.717] | 0.149 |
| L-DLPFC | *suan* (aching) | 0.361 [–0.006, 0.642] | 0.730 | 0.674 [0.343, 0.858] | 0.028* |
| L-DLPFC | *ma* (numbness) | 0.139 [–0.239, 0.481] | 0.730 | 0.003 [–0.431, 0.436] | 0.990 |
| L-DLPFC | *zhang* (fullness) | –0.016 [–0.384, 0.358] | 0.986 | 0.378 [–0.059, 0.700] | 0.196 |
| L-DLPFC | *tong* (dull pain) | –0.150 [–0.487, 0.227] | 0.895 | –0.317 [–0.658, 0.133] | 0.288 |
| L-DLPFC | *zouchuan* (spreading) | 0.127 [–0.252, 0.472] | 0.959 | 0.253 [–0.203, 0.622] | 0.411 |
| L-DLPFC | *zhong* (heaviness) | 0.059 [–0.317, 0.416] | 0.959 | –0.100 [–0.511, 0.343] | 0.769 |
| L-DLPFC | Total *deqi* score | 0.205 [–0.175, 0.530] | 0.730 | 0.375 [–0.063, 0.698] | 0.197 |
| R-DLPFC | *suan* (aching) | 0.331 [–0.040, 0.620] | 0.730 | 0.389 [–0.046, 0.707] | 0.181 |
| R-DLPFC | *ma* (numbness) | 0.153 [–0.224, 0.489] | 0.760 | –0.155 [–0.544, 0.292] | 0.628 |
| R-DLPFC | *zhang* (fullness) | 0.251 [–0.123, 0.564] | 0.730 | 0.464 [0.038, 0.748] | 0.131 |
| R-DLPFC | *tong* (dull pain) | 0.015 [–0.357, 0.384] | 0.730 | –0.393 [–0.710, 0.050] | 0.181 |
| R-DLPFC | *zouchuan* (spreading) | 0.143 [–0.235, 0.483] | 0.959 | 0.177 [–0.273, 0.562] | 0.609 |
| R-DLPFC | *zhong* (heaviness) | 0.227 [–0.151, 0.547] | 0.730 | 0.162 [–0.286, 0.552] | 0.625 |
| R-DLPFC | Total *deqi* score | 0.477 [0.138, 0.714] | 0.378 | 0.362 [–0.079, 0.689] | 0.214 |
| L-SMG | *suan* (aching) | 0.220 [–0.159, 0.542] | 0.730 | 0.531 [0.127, 0.787] | 0.084 |
| L-SMG | *ma* (numbness) | 0.036 [–0.338, 0.401] | 0.961 | –0.282 [–0.632, 0.172] | 0.361 |
| L-SMG | *zhang* (fullness) | –0.094 [–0.443, 0.282] | 0.961 | 0.485 [0.064, 0.761] | 0.131 |
| L-SMG | *tong* (dull pain) | –0.072 [–0.427, 0.306] | 0.961 | –0.253 [–0.612, 0.203] | 0.411 |
| L-SMG | *zouchuan* (spreading) | 0.209 [–0.170, 0.533] | 0.895 | 0.259 [–0.197, 0.626] | 0.411 |
| L-SMG | *zhong* (heaviness) | –0.172 [–0.502, 0.208] | 0.961 | 0.032 [–0.408, 0.462] | 0.922 |
| L-SMG | Total *deqi* score | –0.004 [–0.373, 0.367] | 0.986 | 0.444 [0.015, 0.736] | 0.132 |
| R-SMG | *suan* (aching) | 0.236 [–0.142, 0.555] | 0.730 | 0.432 [–0.001, 0.727] | 0.138 |
| R-SMG | ma (numbness) | 0.199 [–0.181, 0.525] | 0.813 | –0.167 [–0.553, 0.281] | 0.617 |
| R-SMG | *zhang* (fullness) | 0.195 [–0.185, 0.523] | 0.730 | 0.481 [0.060, 0.759] | 0.131 |
| R-SMG | *tong* (dull pain) | –0.253 [–0.562, 0.124] | 0.959 | –0.439 [–0.733, –0.009] | 0.132 |
| R-SMG | *zouchuan* (spreading) | –0.315 [–0.608, 0.055] | 0.730 | 0.311 [–0.138, 0.659] | 0.298 |
| R-SMG | *zhong* (heaviness) | 0.428 [0.077, 0.688] | 0.760 | 0.110 [–0.336, 0.513] | 0.742 |
| R-SMG | Total *deqi* score | 0.314 [–0.056, 0.608] | 0.730 | 0.327 [–0.120, 0.668] | 0.272 |
| L-S1 | *suan* (aching) | 0.411 [0.058, 0.674] | 0.730 | 0.627 [0.271, 0.834] | 0.034* |
| L-S1 | *ma* (numbness) | 0.018 [–0.355, 0.386] | 0.961 | –0.133 [–0.528, 0.314] | 0.688 |
| L-S1 | *zhang* (fullness) | –0.127 [–0.468, 0.252] | 0.959 | 0.560 [0.166, 0.804] | 0.084 |
| L-S1 | *tong* (dull pain) | –0.175 [–0.505, 0.205] | 0.961 | –0.240 [–0.603, 0.217] | 0.426 |
| L-S1 | *zouchuan* (spreading) | 0.044 [–0.331, 0.408] | 0.959 | 0.253 [–0.203, 0.622] | 0.411 |
| L-S1 | *zhong* (heaviness) | –0.076 [–0.430, 0.303] | 0.933 | –0.131 [–0.526, 0.316] | 0.688 |
| L-S1 | Total *deqi* score | 0.028 [–0.345, 0.394] | 0.730 | 0.535 [0.133, 0.789] | 0.084 |
| R-S1 | *suan* (aching) | 0.251 [–0.123, 0.564] | 0.730 | 0.441 [0.010, 0.735] | 0.132 |
| R-S1 | *ma* (numbness) | 0.019 [–0.354, 0.387] | 0.961 | –0.013 [–0.442, 0.421] | 0.965 |
| R-S1 | *zhang* (fullness) | –0.188 [–0.514, 0.191] | 0.803 | 0.563 [0.170, 0.805] | 0.084 |
| R-S1 | *tong* (dull pain) | 0.030 [–0.343, 0.396] | 0.959 | –0.473 [–0.754, –0.050] | 0.131 |
| R-S1 | *zouchuan* (spreading) | 0.057 [–0.319, 0.414] | 0.959 | 0.238 [–0.219, 0.612] | 0.426 |
| R-S1 | *zhong* (heaviness) | –0.218 [–0.538, 0.158] | 0.959 | 0.057 [–0.387, 0.453] | 0.868 |
| R-S1 | Total *deqi* score | –0.105 [–0.451, 0.272] | 0.959 | 0.483 [0.061, 0.760] | 0.131 |
| L-SAC | *suan* (aching) | 0.311 [–0.058, 0.606] | 0.730 | 0.732 [0.441, 0.886] | 0.028* |
| L-SAC | ma (numbness) | 0.279 [–0.097, 0.584] | 0.730 | –0.365 [–0.689, 0.078] | 0.213 |
| L-SAC | *zhang* (fullness) | –0.049 [–0.413, 0.327] | 0.961 | 0.633 [0.280, 0.837] | 0.034* |
| L-SAC | *tong* (dull pain) | –0.017 [–0.385, 0.357] | 0.961 | –0.077 [–0.494, 0.366] | 0.807 |
| L-SAC | *zouchuan* (spreading) | 0.061 [–0.315, 0.418] | 0.961 | 0.239 [–0.218, 0.613] | 0.426 |
| L-SAC | *zhong* (heaviness) | –0.012 [–0.381, 0.362] | 0.959 | –0.242 [–0.614, 0.214] | 0.426 |
| L-SAC | Total *deqi* score | 0.283 [–0.093, 0.587] | 0.730 | 0.597 [0.221, 0.824] | 0.056 |
| R-SAC | *suan* (aching) | 0.154 [–0.223, 0.490] | 0.875 | 0.419 [–0.016, 0.718] | 0.146 |
| R-SAC | *ma* (numbness) | 0.182 [–0.198, 0.512] | 0.745 | 0.155 [–0.293, 0.546] | 0.628 |
| R-SAC | *zhang* (fullness) | 0.059 [–0.317, 0.416] | 0.961 | 0.541 [0.142, 0.793] | 0.084 |
| R-SAC | *tong* (dull pain) | –0.045 [–0.410, 0.331] | 0.961 | –0.462 [–0.748, –0.036] | 0.131 |
| R-SAC | *zouchuan* (spreading) | –0.227 [–0.545, 0.148] | 0.760 | 0.077 [–0.370, 0.493] | 0.807 |
| R-SAC | *zhong* (heaviness) | 0.256 [–0.118, 0.567] | 0.730 | –0.077 [–0.494, 0.366] | 0.807 |
| R-SAC | Total *deqi* score | 0.227 [–0.151, 0.547] | 0.730 | 0.443 [0.013, 0.735] | 0.132 |

Note: *rho*, Spearman correlation coefficient, with its 95% CI estimated using Fisher’s z transformation. *Deqi* items: Total *deqi* score; *suan*, aching or soreness; *zhang*, fullness/distention or pressure; *ma*, numbness or tingling; *tong*, dull pain; *zhong*, heaviness; *zouchuan*, spreading. All *p*-values were corrected using the FDR method. ROI abbreviations are defined in Table 1. **p* < 0.05.
